# Association Mapping and Haplotype Analysis of a 3.1-Mb Genomic Region Involved in Fusarium Head Blight Resistance on Wheat Chromosome 3BS

**DOI:** 10.1371/journal.pone.0046444

**Published:** 2012-10-05

**Authors:** Chenyang Hao, Yuquan Wang, Jian Hou, Catherine Feuillet, Francois Balfourier, Xueyong Zhang

**Affiliations:** 1 Key Laboratory of Crop Gene Resources and Germplasm Enhancement, Ministry of Agriculture/The National Key Facility for Crop Gene Resources and Genetic Improvement/Institute of Crop Science, Chinese Academy of Agricultural Sciences, Beijing, China; 2 INRA UMR 1095, Genetics Diversity and Ecophysiology of Cereals, Clermont-Ferrand, France; Nanjing Agricultural University, China

## Abstract

A previous study provided an in-depth understanding of molecular population genetics of European and Asian wheat gene pools using a sequenced 3.1-Mb contig (*ctg954*) on chromosome 3BS. This region is believed to carry the *Fhb1* gene for response to Fusarium head blight. In this study, 266 wheat accessions were evaluated in three environments for Type II FHB response based on the single floret inoculation method. Hierarchical clustering (UPGMA) based on a Manhattan dissimilarity matrix divided the accessions into eight groups according to five FHB-related traits which have a high correlation between them; Group VIII comprised six accessions with FHB response levels similar to variety Sumai 3. Based on the compressed mixed linear model (MLM), association analysis between five FHB-related traits and 42 molecular markers along the 3.1-Mb region revealed 12 significant association signals at a threshold of *P*<0.05. The highest proportion of phenotypic variation (6.2%) in number of diseased spikelets (NDS) occurred at locus cfb6059, and the physical distance was about 2.9 Kb between umn10 and this marker. Haplotype block (HapB) analysis using a sliding window LD of 5 markers, detected six HapBs in the 3.1-Mb region at *r^2^*>0.1 and *P*<0.001 between random closely linked markers. *F*-tests among Haps with frequencies >0.05 within each HapB at *r^2^*>0.1 and *P*<0.001 showed significant differences between the Hap carried by FHB resistant resources, such as Sumai 3 and Wangshuibai, and susceptible genotypes in HapB3 and HapB6. These results suggest that *Fhb1* is located within HapB6, with the possibility that another gene is located at or near HapB3. SSR markers and Haps detected in this study will be helpful in further understanding the genetic basis of FHB resistance, and provide useful information for marker-assisted selection of *Fhb1* in wheat breeding.

## Introduction

Fusarium head blight (FHB), or scab, caused mainly by *Fusarium graminearum* Schwabe (*Giberella zeae* Schw. and Petch), is a devastating worldwide fungal disease of wheat (*Triticum aestivum* L.). FHB infection is favored by warm humid conditions during the flowering and early stages of kernel development [1]. FHB reduces yield and grain quality through shrivelled kernels, and contaminates the grains with mycotoxins such as deoxynivalenol [1]–[4] making the grains unsuitable for human or animal consumption [5].

The use of host resistance is an economically and environmentally effective strategy for controlling FHB. So far, only a few highly resistant wheat cultivars have been identified from different geographic regions, including Asia, South and North America, and Europe [6]–[8]; for example, spring wheats from Asia including Sumai 3 (Funo/Taiwan Wheat) and its derivatives, spring wheats from Brazil and winter wheats from Europe. However, wheat breeding programs worldwide have relied heavily on Sumai 3-derived FHB resistance with a risk of rapid overcome of the resistance. The utilization of novel resistance sources is needed to diversify the genetic basis of FHB resistance and to increase the level of resistance through pyramiding of resistance genes that tend to act additively [9].

During the past decade, numerous studies in wheat have focused on molecular mapping FHB resistan*ce* through linkage analysis [7], [10]–[15]. From 52 studies on genetic mapping populations, more than 100 quantitative trait loci (QTL) for FHB resistance were identified on all wheat chromosomes except chromosome 7D [16], [17]. The FHB resistance loci that were fine mapped using similar strategies include *Fhb1* [13], [18], *Fhb2* [19], *Fhb4* [20] and *Fhb5* [15]. Among them the major QTL designated as *Fhb1* (syn. *Qfhs.ndsu-3BS*) that derived from Sumai 3 was originally identified by RFLP analysis in a recombinant inbred population [21] and later confirmed in numerous studies [7], [10], [11], [13], [18], [22]–[25]. This FHB resistance gene was originally located in the distal region of chromosome 3BS between SSR loci *gwm493* and *gwm533* [10]. Several DNA markers were developed in this chromosome region in subsequent studies, including *sts3B.189*, *sts3B.206* [18] and *UMN10* [26], suitable for marker-assisted selection for gene *Fhb1*. Almost all the markers were identified in the bi-parental populations through linkage mapping.

Association mapping, a powerful approach to unravel the genetic architecture of complex traits in crops [27], [28], has been used in few studies to identify the relatedness between molecular markers and FHB resistance in wheat natural populations [29], [30]. Moreover, haplotype association is likely to be more powerful in the presence of LD [31]. A haplotype is a set of closely linked intra-chromosome genetic markers that tend to be inherited together [32]. Following genetic diversity and linkage disequilibrium studies on a 3.1-Mb genomic region on chromosome 3B in European and Asian bread wheat populations [33], the same set of accessions were verified on FHB-related traits in multiple environments. In this paper, our objective, as in the previous study, was to target loci significantly associated with *Fhb1* in European and Asian wheats through association analysis in a candidate region in contig *ctg954* (Genebank accession number: FN564434) on the short arm of chromosome 3B. Haplotype diversity and its relationship to FHB-related traits were also analyzed in the 3.1-Mb genomic region. Such an association analysis should provide useful information for marker-assisted selection of *Fhb1* in wheat breeding. The ultimate aim in the study was to discover potential resistance sources for use in wheat breeding and genetic improvement.

## Results

### Genetic Structure and Relative Kinship of Overall Wheat Accessions

**Figure 1:**
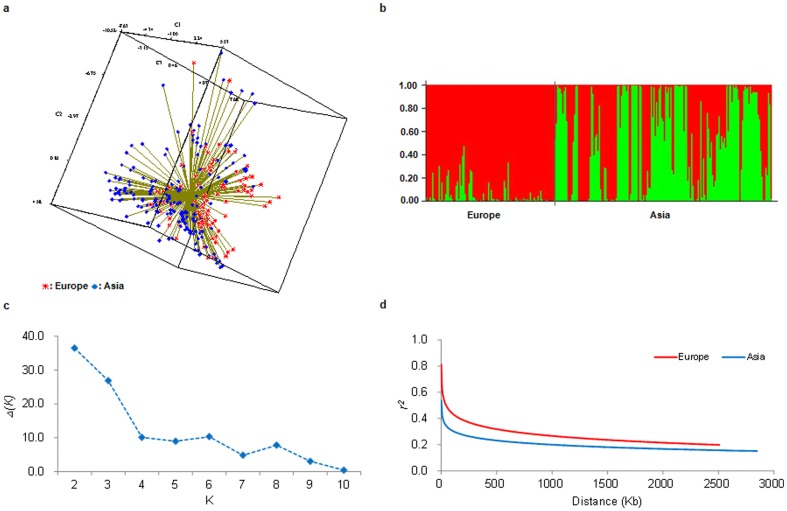
Analysis of genetic relationships, population structure and LD decay in European and Asian wheat accessions. (a) Principal coordinate analysis of European (red) and Asian (blue) accessions. (b) Bayesian clustering (STRUCTURE, K = 2) of wheat accessions. (c) Estimation of the number of populations by calculating delta K values. (d) Average LD decay in European (red) and Asian (blue) accessions.

Based on genotyping datasets of 70 genome-wide microsatellites, principal coordinate analysis of the 266 accessions revealed that the European and the Asian materials were independent sub-groups (Figure 1a). Re-evaluation of genetic structure for the test genotypes with STRUCTURE software further demonstrated two sub-groups, as revealed by the Evano criterion (Figure 1b, c). Figure 1d indicates that average LD decay could be higher than 500 Kb with *r^2^*<0.2 at *P*<0.001 along the 3.1-Mb region for the two sub-groups. The European sub-group had stronger LD than the Asian sub-group. These results were consistent with the previous study on 376 accessions using the same genotyping dataset [33].

**Figure 2:**
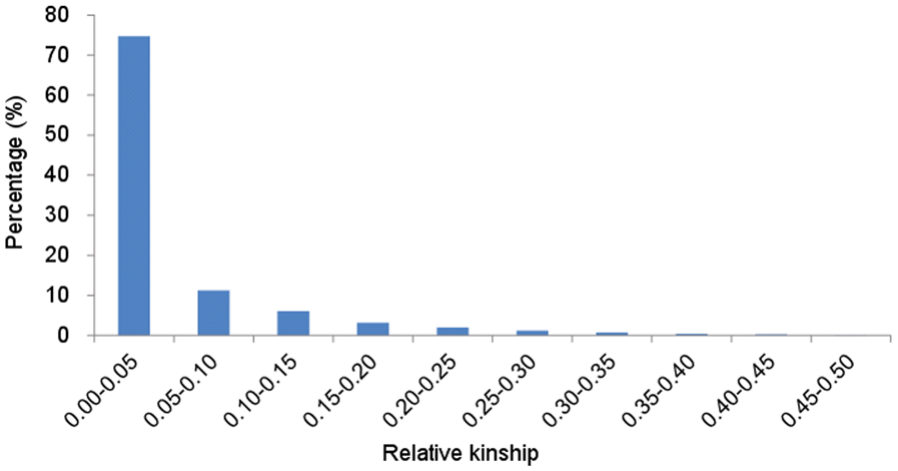
Distribution of pair-wise kinship coefficients among 266 bread wheat accessions based on 70 whole genome SSR markers.

Besides evaluation of genetic structure of the test genotypes based on the 70 genome-wide SSRs with major allelic frequencies (MAF) >5% at the population level, the same sets of genotyping data were used to calculate relative kinship between pairs of individuals in the current study. This revealed the approximate identity between two given individuals over the average probability of identity between any two random individuals [34], [35]. About 74.7% of the pairwise kinship estimates ranged from 0 to 0.05, among which the percentage of kinship values close to 0 reached as high as 57.1% (Figure 2). This indicated unrelated of the accessions. Other ranges of pair-wise kinships showed an obviously declining tendency.

### FHB Responses in Three Environments

Using the phenotypic data from three environments and their average values, comparisons were performed across environments for each FHB-related trait (Table 1). Obvious differences were observed for each of the five FHB-related traits in all environments. For example, in E1, the ranges of NDS, PDS, LDR, DS and DI were 1.00 to 8.53, 4.21 to 41.51, 0 to 6.97, 0 to 0.76 and 0 to 22.66, respectively. The same inner variances were also present in other environments. Highly consistent results were obtained among the three environments. No statistically significant difference was detected in most tests, except between E1 and the others in PDS and LDR. Furthermore, FBH-resistance-related traits of the four control accessions also showed good consistency among the three environments (Table S1). With the help of phenotypic data and the kinship matrix, heritability (*h^2^*) was calculated for each trait (Table 1). The *h^2^* ranged from 56.5% to 62.5% using average values of the three environments. The highest *h^2^* value was for DI (62.5%), indicating that this trait was less affected by environmental factors than the other four.

**Table 1:** Comparisons of five FHB-related traits in three environments.

Trait	E1			E2			E3			Total		
	Mean±S.E	Range	*h^2^* (%)*	Mean±S.E	Range	*h^2^* (%)	Mean±S.E	Range	*h^2^* (%)	Mean±S.E	Range	*h^2^* (%)
NDS	4.42±0.11a	1.00–8.53	52.1	4.44±0.11a	1.00–10.20	51.4	4.66±0.07a	1.00–7.00	55.8	4.50±0.08a	1.03–7.58	60.4
PDS	20.73±0.50a	4.21–41.51	53.5	22.85±0.60b	4.55–50.00	52.4	22.67±0.36b	6.46–39.56	61.4	22.08±0.42ab	6.09–39.56	61.1
LDR	3.79±0.080a(A)	0–6.97	55.0	3.53±0.08b(AB)	0.05–6.51	42.4	3.45±0.04b(B)	0.93–5.57	48.7	3.59±0.06ab(AB)	0.38–5.78	56.5
DS	0.34±0.01a	0–0.76	58.8	0.34±0.01a	0.01–0.65	45.3	0.35±0.00a	0.10–0.52	52.8	0.34±0.01a	0.05–0.57	60.9
DI	8.00±0.30a	0–22.66	54.4	8.83±0.37a	0.02–27.61	48.2	8.18±0.20a	0.74–20.90	66.3	8.34±0.25a	0.30–20.28	62.5

Note: Capital and small letters show the significance level at *P*<0.01 and *P*<0.05 compared between environments for the same trait, respectively.

NDS: Number of diseased spikelets; PDS: Percentage of diseased spikelets; LDR: Length of diseased richides; DS: Disease severity; DI: Disease index.

NAU: Nanjing Agricultural University; JAAS: Jiangsu Academy of Agricultural Sciences; E1: NAU_2009; E2: JAAS_2009; E3: NAU_2010.

* Heritability.

Pearson correlation coefficients between environments were calculated for each FHB-related trait using SPSS v12.0 (Table 2). There were highly significant positive correlations between environments for all five traits. The ranges in the environmental correlation coefficients without taking into account mean value for NDS, PDS, LDR, DS and DI were 0.498 to 0.676, 0.539 to 0.699, 0.530 to 0.675, 0.531 to 0.676 and 0.542 to 0.689, respectively. Moderately high correlations between environments indicated good repetitiveness and high heritability in the selected population. It was notable that the coefficients between the average and each environment were relatively higher than that between individual environments, indicating that the average value had a high representation in the test traits. Pearson correlation coefficients between traits were therefore estimated using the average values of the three environments (Table S2). There were significant (*P*<0.01) positive correlations between all FHB resistance traits. All pairs of phenotypic correlation coefficients ranged from 0.778 to 0.970, and the highest value was observed for the correlation between the PDS and DI. This was consistent with the interrelationship among these parameters at the statistical level.

**Table 2:** Correlation analyses of three environments using mean values of five FHB-related traits.

	NDS				PDS				LDR				DS				DI			
	E1	E2	E3	T	E1	E2	E3	T	E1	E2	E3	T	E1	E2	E3	T	E1	E2	E3	T
E1	1	0.498**	0.676**	0.850**	1	0.539**	0.687**	0.843**	1	0.530**	0.675**	0.873**	1	0.531**	0.676**	0.867**	1	0.542**	0.667**	0.839**
E2		1	0.655**	0.856**		1	0.699**	0.883**		1	0.644**	0.852**		1	0.656**	0.859**		1	0.689**	0.889**
E3			1	0.867**			1	0.884**			1	0.852**			1	0.858**			1	0.870**
T				1				1				1				1				1

** Significance at *P*<0.01.

T: total; E1, E2, E3 show different environments as in Table 1.

NDS: Number of diseased spikelets; PDS: Percentage of diseased spikelets; LDR: Length of diseased richides; DS: Disease severity; DI: Disease index.

### Hierarchical Clustering of FHB Traits

In order to infer possible classifications for the test genotypes, hierarchical clustering (UPGMA) of all wheat accessions based on Manhattan dissimilarity matrix with DARwin v5 software using the five FHB-related traits was used (Figure S1). The hierarchical tree showed that the genotypes could be divided into eight groups. As shown in Figure S1, the numbers of genotypes in each group were 51, 20, 51, 53, 28, 37, 20 and 6. The resistant controls, Sumai 3 and Wangshuibai, clustered with group VIII, whereas the susceptible controls, Ningmai 11 and Mianyang 11, were in groups III and VI, respectively.

A comparative analysis of the five traits between clusters was made based on the average values for the three environments (Table 3). Cluster VIII had the lowest mean values for all five traits, whereas II had the highest. This indicated that accessions in cluster VIII were the most resistant among the eight subgroups. The order of clusters, from resistant to susceptible, was the same for all five traits, i.e. VIII, VII, V, VI, III, IV, I, II. Significant differences between clusters further revealed that subgroup VIII, including the two positive controls, had significant (*P*<0.01) resistance compared to the other subgroups, indicating that these six accessions might therefore have potential as resistant genetic resources for improvement of FHB resistance in wheat breeding around the world. Detailed information for the six accessions in subgroup VIII is provided in Table S3.

**Table 3:** Comparisons of eight groups based on hierarchical clustering using five FHB-related traits.

Cluster	Number	NDS		PDS		LDR		DS		DI	
		Mean±S.E	Range	Mean±S.E	Range	Mean±S.E	Range	Mean±S.E	Range	Mean±S.E	Range
I	51	5.84±0.07A	4.89–6.94	29.15±0.23A	26.57–33.01	4.33±0.08a(A)	3.01–5.78	0.42±0.01A	0.35–0.51	12.47±0.18a(A)	9.78–15.31
II	20	6.47±0.11B	5.67–7.58	34.26±0.44B	31.27–39.56	4.40±0.16a(A)	2.50–5.53	0.47±0.01B	0.41–0.57	16.38±0.32b(B)	13.73–20.28
III^a^	51	4.48±0.06C	3.41–5.46	21.30±0.16C	19.07–23.17	3.75±0.07b(B)	2.45–4.94	0.34±0.01C	0.22–0.43	7.51±0.13c(C)	5.51–8.97
IV	53	4.96±0.07D	3.65–6.10	24.61±0.13D	22.37–26.39	3.98±0.06b(AB)	3.09–4.74	0.39±0.01D	0.30–0.47	9.62±0.12d(D)	7.54–11.99
V	28	3.11±0.04E	2.58–3.62	14.32±0.13E	13.06–15.36	2.79±0.10c(C)	1.94–4.06	0.25±0.01E	0.20–0.38	3.84±0.13e(E)	2.61–5.77
VI^b^	37	3.78±0.06F	3.06–4.50	17.66±0.15F	15.95–19.15	3.19±0.07d(C)	2.23–3.90	0.29±0.01F	0.20–0.39	5.41±0.14f(F)	3.51–7.19
VII	20	2.25±0.08G	1.58–2.96	10.81±0.30G	8.19–12.30	2.01±0.12e(D)	1.08–2.92	0.20±0.01G	0.10–0.25	2.42±0.16g(G)	1.17–3.66
VIII^c^	6	1.30±0.10H	1.03–1.72	6.86±0.28H	6.09–7.91	1.15±0.26f(E)	0.38–2.08	0.12±0.02H	0.05–0.18	0.86±0.16h(G)	0.30–1.36

a Includes negative control Ningmai 11;

b Includes negative control Mianyang 11;

c Including positive controls Sumai 3 and Wangshuibai. Capital and small letters show significance at *P*<0.01 and *P*<0.05 between clusters for the same trait, respectively.

NDS: Number of diseased spikelets; PDS: Percentage of diseased spikelets; LDR: Length of diseased richides; DS: Disease severity; DI: Disease index.

### Association Studies for Five FHB Traits along the 3.1-Mb Region

**Figure 3:**
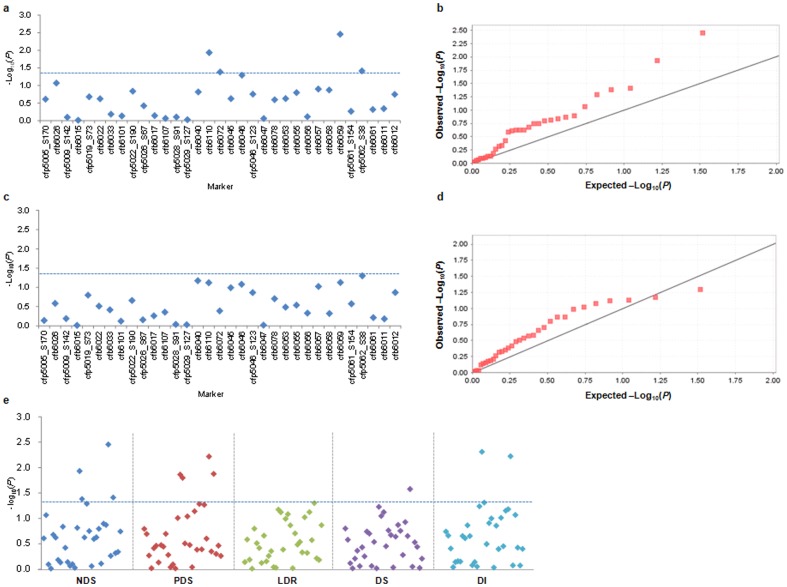
Associations of five FHB-related traits with molecular markers in the 3.1 -Mb genomic region. (a) Dot plots of the compressed mixed linear model (MLM) for numbers of diseased spikelets (NDS). Negative log_10_-transformed *P* values in a sequenced contig (*ctg954*) of 3.1-Mb are plotted against position along the chromosome region. Blue horizontal dashed line represents the chromosome-region significance threshold. (b) Quantile-quantile plot of compressed MLM for NDS. (c) Dot plots of compressed MLM for lengths of diseased rachides (LDR), as in a. (d) Quantile-quantile plot of compressed MLM for LDR. (e) Dot plots of compressed MLM for five FHB phenotypic traits, i.e. number of diseased spikelets (NDS), percentage of diseased spikelets (PDS), the length of diseased rachides (LDR), disease severity (DS) and disease index (DI), as in a.

Given the strong population clustering of the test genotypes, the compressed mixed linear model (MLM) [36], [37] of the Q+K model was used to identify association signals related to the five FHB-related traits using mean values of three environments, viz. number of diseased spikelets (NDS), percentage of diseased spikelets (PDS), length of diseased rachides (LDR), disease severity (DS) and disease index (DI), with 42 molecular markers (32 SSR and 10 SNP) along the 3.1-Mb region on chromosome 3BS. The results of the association studies, including the Manhattan and quantile-quantile plots, are mapped in the Figure 3 and Figure S2. Twelve significant association signals were found at the threshold of *P*<0.05 (Table 4). No significant association was identified for LDR. The number of significant associations was 4, 4, 1 and 3 for NDS, PDS, DS and DI, respectively. Among the 12 associations, locus *cfb6059* accounted for the highest amount of phenotypic variation (6.2%) for NDS, whereas *cfp5062_S38* explained the lowest (1.6%). Most markers had significant associations with traits in a specific environment, and only three significant associations (between *cfb6110* and DI, between *cfb6072* and PDS, and between *cfb6072* and DI) occurred in two environments. In total, four loci including three SSR (*cfb6110*, *cfb6072* and *cfb6059*) and one SNP (*cfp5062_S38*) were significantly associated with four FHB-related traits, and for each locus, there were at least two significantly associated traits. Locus *cfb6059* was associated with four traits, indicating the relatedness between the traits or a situation of multiple traits determined by one gene.

**Table 4:** Significance (*P* value) and genetic variation (*R^2^*) explained by individual markers associated with FHB-related traits.

Trait	cfb6110		cfb6072		cfb6059		cfp5062_S38	
	*P* value	*R^2^* (%)	*P* value	*R^2^* (%)	*P* value	*R^2^* (%)	*P* value	*R^2^* (%)
NDS	0.012*	2.8	0.042*	4.8	0.004**	6.2	0.039*	1.6
PDS	0.014*	2.7	0.016*	5.9	0.006**	5.6	0.013*	2.4
E1_PDS_			0.005**	6.8				
E3_PDS_			0.024*	5.3				
DS					0.027*	4.3		
DI	0.005**	3.5	0.049*	4.8	0.006**	5.7		
E1_DI_			0.015*	5.9				
E2_DI_	0.013*	2.8						
E3_DI_	0.026*	2.2	0.034*	5.1				

* : *P*<0.05;

** : *P*<0.01.

NDS: Number of diseased spikelets; PDS: Percentage of diseased spikelets; DS: Disease severity; DI: Disease index.

E1: NAU_2009; E2: JAAS_2009; E3: NAU_2010.

**Figure 4:**
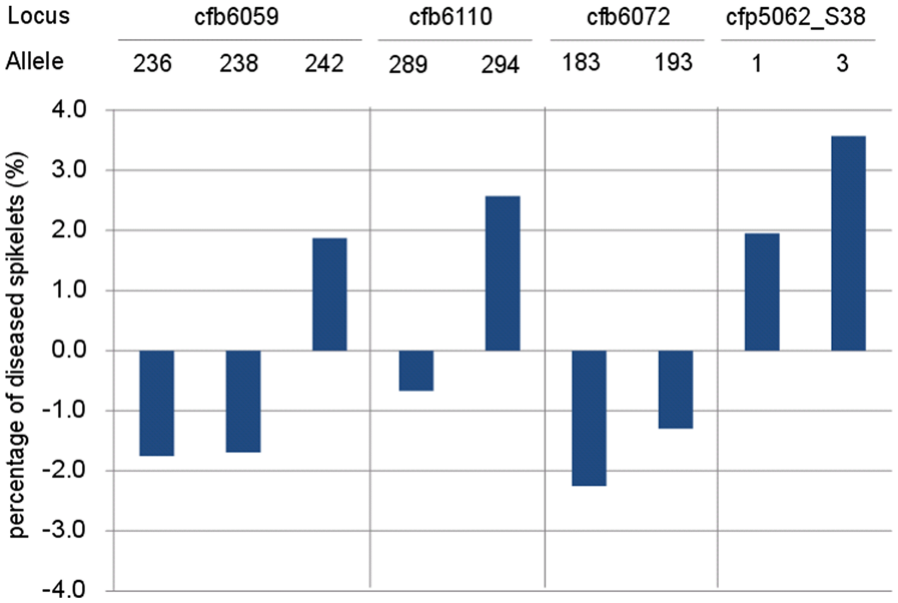
Comparison of allelic effects of four significantly associated loci for PDS (%).

After detection of molecular markers significantly associated with FHB response traits, the mean phenotypic values and genetic effects of alleles with frequencies of >0.05 for the four significantly associated markers were determined (Table 5). There were no significant phenotypic differences in mean values for the five scab traits between two random alleles at both the *cfb6059* and *cfp5062_S38* loci. Significant phenotypic differences (*P*<0.05) were found between the two alleles at *cfb6072* only for DS. However, at *cfb6110*, significant differences (*P*<0.01) were detected between alleles *cfb6110_289_* and *cfb6110_294_* for traits NDS, PDS, LDR and DI. Accessions with *cfb6110_289_* included Sumai 3 and those sharing the same Hap with Sumai 3 had lower mean phenotypic values than those with *cfb6110_294_* for all four traits. Taking PDS as a trait example (Figure 4), there were negative genetic effects for the alleles *236 bp* and *238 bp* at *cfb6059*, *289 bp* at *cfb6110*, *183 bp* and *193 bp* at *cfb6072*, and positive effects for *242 bp* at *cfb6059*, *294 bp* at *cfb6110*, and alleles *1* and *3* at *cfp5062_S38*. Accessions with negative alleles had relatively higher FHB resistance than those with positive alleles. Similar allelic effects were detected for the other four FHB-related traits (Figure S3).

**Table 5:** Genetic effects of major alleles (frequency >0.05) for four markers significantly associated with five FHB-related traits.

Trait	cfb6059				cfb6110				cfb6072				cfp5062_S38			
	Allele (bp)	F (%)	Mean±S.E	Allele effect	Allele (bp)	F (%)	Mean±S.E	Allele effect	Allele (bp)	F (%)	Mean±S.E	Allele effect	Allele (bp)	F (%)	Mean±S.E	Allele effect
NDS	236	72.6	4.43±0.09a	−0.37	289*	57.5	4.31±0.11A	−0.16	183*	41.7	4.36±0.13a	−0.49	1	69.2	4.43±0.09a	0.58
	238*	10.5	4.40±0.30a	−0.39	294	24.4	4.99±0.15B	0.52	193	37.2	4.46±0.13a	−0.39	3*	29.3	4.72±0.18a	0.87
	242	6.0	5.06±0.24a	0.27												
PDS	236	72.6	21.67±0.46a	−1.75	289*	57.5	21.17±0.56A	−0.67	183*	41.7	21.09±0.69a	−2.25	1	69.2	21.64±0.46a	1.95
	238*	10.5	21.73±1.61a	−1.69	294	24.4	24.41±0.80B	2.57	193	37.2	22.32±0.67a	−1.30	3*	29.3	23.26±0.91a	3.57
	242	6.0	25.29±1.49a	1.87												
LDR	236	72.6	3.58±0.07a	0.03	289*	57.5	3.44±0.08A	−0.18	183*	41.7	3.43±0.09a	−0.38	1	69.2	3.60±0.06a	0.43
	238*	10.5	3.45±0.20a	−0.10	294	24.4	3.90±0.10B	0.28	193	37.2	3.64±0.09a	−0.18	3*	29.3	3.58±0.13a	0.41
	242	6.0	3.96±0.17a	0.40												
DS	236	72.6	0.34±0.01a	−0.01	289*	57.5	0.34±0.01a	−0.01	183*	41.7	0.33±0.01a	−0.03	1	69.2	0.34±0.01a	0.01
	238*	10.5	0.33±0.02a	−0.03	294	24.4	0.37±0.01a	0.03	193	37.2	0.36±0.01b	−0.01	3*	29.3	0.35±0.01a	0.02
	242	6.0	0.38±0.02a	0.03												
DI	236	72.6	8.09±0.28a	−1.25	289*	57.5	7.82±0.32A	−0.46	183*	41.7	7.62±0.39a	−1.58	1	69.2	8.08±0.28a	0.60
	238*	10.5	8.01±0.84a	−1.33	294	24.4	9.60±0.52B	1.32	193	37.2	8.65±0.40a	−0.55	3*	29.3	9.00±0.52a	1.52
	242	6.0	10.13±0.93a	0.79												

* Includes the allele carried by Sumai 3 at each locus. Capital and small letters show significance at *P*<0.01 and *P*<0.05 when comparing alleles at the same locus for each trait.

F: Frequency; NDS: Number of diseased spikelets; PDS: Percentage of diseased spikelets; LDR: Length of diseased richides; DS: Disease severity; DI: Disease index.

### Haplotypes of 42 Markers along the 3.1-Mb Region and Their Phenotypic Effects

**Figure 5:**
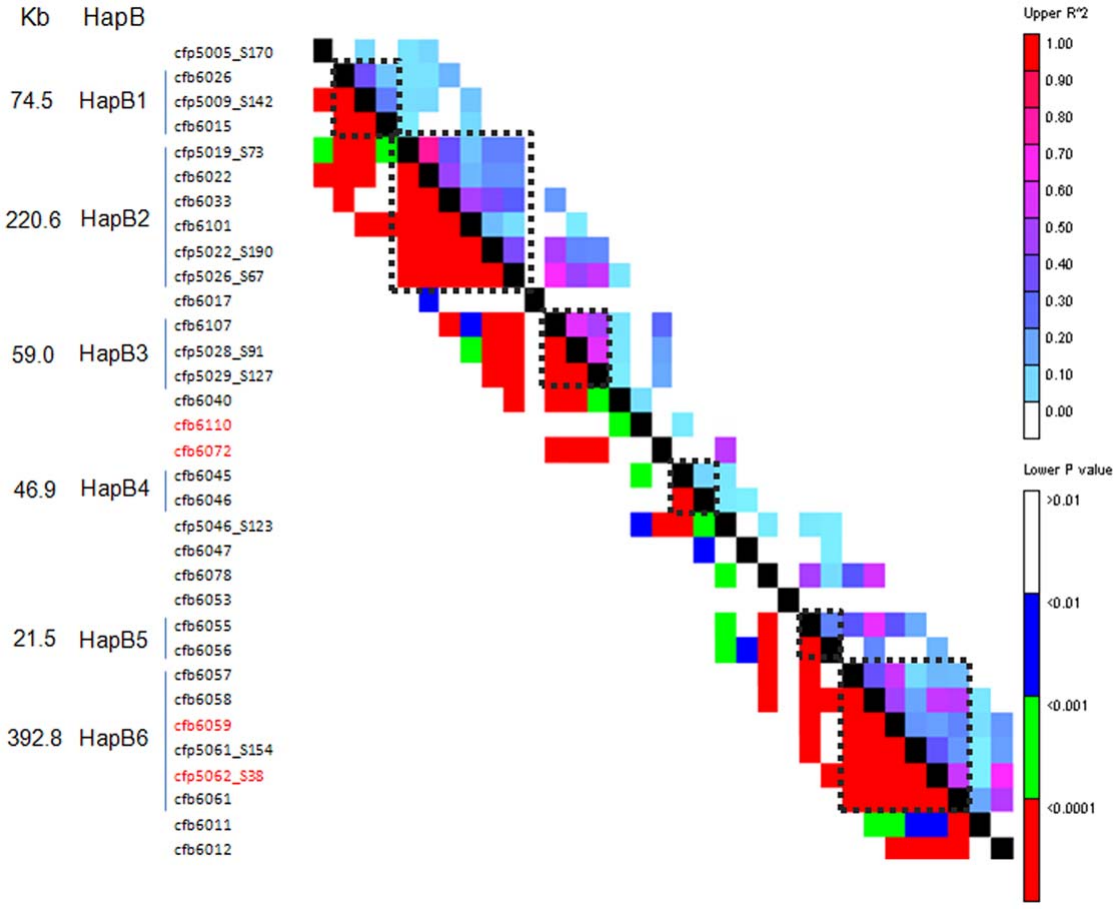
Haplotype blocks (HapB) and physical distances (Kb) defined by sliding window LD of 5 marker sets at the level of *r^2^*>0.1 and *P*<0.001. Red markers are those significantly associated with scab-related traits in this study.

To decide the number of haplotype blocks (HapB) for 42 markers along the contig *ctg954*, HapB analysis was conducted by sliding window LD with 5 marker sets as the LD window size using TASSEL v3.0 software [37]. Six HapBs were found at *r^2^*>0.1 and *P*<0.001 between random closely linked markers in the sliding window LD (Figure 5, Table 6). As shown in Table 6, the numbers of markers constituting HapBs varied from 2 to 6, and physical distances ranged from 21.5 Kb (HapB5) to 392.8 Kb (HapB6). Based on the allelic combinations for different markers in each HapB, the numbers of expected Haps for HapB1 to HapB6 were 24, 96, 16, 35, 4, and 1,584. However, the observed frequencies were 14, 21, 10, 19, 4 and 32, respectively. Therefore, except for HapB5, there were many fewer Haps than expected. Moreover, the frequency of Haps was also different within each HapB, and the dominant Hap frequencies for HapB1 to HapB6 were 39.7%, 29.0%, 59.9%, 30.2%, 58.2% and 70.6%, respectively. There was a large change in the Hap frequency for HapB5, the highest frequency of the major Hap reached 58.2%, although the observed and expected Hap numbers were consistent (Table 6).

**Table 6:** Haplotype information including number of markers, physical distance (Kb), the expected and observed haplotypes and frequency of major haplotype for each haplotype block with *r^2^*>0.1 and *P*<0.001.

Haplotype block (HapB)	No. of markers	Name of marker	Position (bp)	No. of alleles	Distance (Kb)	Expected Hap^a^	Observed Hap	No. of accessions^b^	% of major Hap
HapB1	3	cfb6026	237882	2	74.5	24	14	194	39.7
		cfp5009_S142	287416	2					
		cfb6015	312390	6					
HapB2	6	cfp5019_S73	730302	2	220.6	96	21	210	29.0
		cfb6022	738844	2					
		cfb6033	767663	3					
		cfb6101	791946	2					
		cfp5022_S190	814617	2					
		cfp5026_S67	950903	2					
HapB3	3	cfb6107	996915	4	59.0	16	10	172	59.9
		cfp5028_S91	1031758	2					
		cfp5029_S127	1055910	2					
HapB4	2	cfb6045	1705543	5	46.9	35	19	252	30.2
		cfb6046	1752462	7					
HapB5	2	cfb6055	2392946	2	21.5	4	4	251	58.2
		cfb6056	2414424	2					
HapB6	6	cfb6057	2501231	3	392.8	1584	32	194	70.6
		cfb6058	2517088	11					
		cfb6059	2517764	3					
		cfp5061_S154	2633155	2					
		cfp5062_S38	2663984	2					
		cfb6061	2894049	4					

a The number of expected Hap is calculated based on the allelic combinations for different markers in each HapB.

b Accessions without missing data for all markers in each haplotype block.

Hap: haplotype.

Based on the observed Haps within each HapB at *r^2^*>0.1 and *P*<0.001, those Haps with frequencies >0.05 in the studied population, i.e. 6 in HapB1, 6 in HapB2, 3 in HapB3, 4 in HapB4, 3 in HapB5, 2 in HapB6, were selected for tests of association with the five FHB-related traits (Table 7). The Haps carried by Sumai 3 were also included in the comparative analysis (asterisked in Table 7), although its frequency in HapB6 was less than 0.05. Almost no significance was detected between Haps in HapB1 and HapB5. For HapB2 and HapB4, there were some significant signals between Haps, but not necessarily between the Sumai 3 Hap and the others. Statistical significances in the levels of *P* = 0.05 or *P* = 0.01 between the Sumai 3 Hap and the others in the HapB3 and HapB6 regions, except the trait disease index (DI) for HapB3 were very clear. Moreover, the accessions with HapB6-2 and HapB3-2, such as Sumai 3, had the lowest values for the five FHB-related traits. Therefore, a genetic effect analysis of haplotype combinations of HapB3 and HapB6 was performed for the five FHB traits (Table 8). There were six combinations between the two HapBs, and statistical significances at *P*<0.05 or lower occurred in all HapB3-2/HapB6-2 combinations. However, there were no statistical differences among the other five combinations. This indicated that there might be strong interactions between haplotypes HapB3-2 and HapB6-2 leading to increased FHB resistance.

**Table 7:** FHB-related values and *F*- tests among haplotypes with the frequencies >0.05 within each haplotype block with *r^2^*>0.1 and *P*<0.001.

HapB code	No. of Haps	Hap code	Frequency (%)	NDS		PDS		LDR		DS		DI	
				Mean±S.E	*F* test	Mean±S.E	*F* test	Mean±S.E	*F* test	Mean±S.E	*F* test	Mean±S.E	*F* test
HapB1	6	HapB1-1	7.7	3.63±0.24	a	17.62±1.38	a	3.09±0.23	a	0.29±0.02	a	5.86±0.76	a
		HapB1-2	11.3	4.20±0.24	a	20.48±1.20	a	3.54±0.17	a	0.33±0.02	a	7.27±0.69	a
		HapB1-3*	9.8	3.95±0.42	a	20.23±2.27	a	3.22±0.34	a	0.31±0.03	a	7.64±1.18	a
		HapB1-4	9.8	4.68±0.27	a	22.54±1.54	a	3.75±0.21	a	0.34±0.02	a	8.44±1.02	a
		HapB1-5	39.7	4.58±0.12	a	22.62±0.68	a	3.68±0.08	a	0.35±0.01	a	8.47±0.41	a
		HapB1-6	8.8	4.62±0.39	a	22.44±1.87	a	3.66±0.27	a	0.35±0.02	a	8.46±0.94	a
HapB2	6	HapB2-1*	17.6	4.10±0.31	a	20.31±1.57	a	3.05±0.21	a	0.31±0.02	a	7.48±0.84	a
		HapB2-2	7.6	4.17±0.31	ab	20.39±1.64	ab	3.47±0.25	ab	0.32±0.02	abc	7.10±0.98	abd
		HapB2-3	6.2	5.32±0.24	b	27.10±1.43	b	4.04±0.20	b	0.40±0.02	bc	11.27±0.89	c
		HapB2-4	29.0	4.41±0.14	ab	20.90±0.69	a	3.63±0.11	b	0.33±0.01	ab	7.48±0.39	ab
		HapB2-5	9.0	4.94±0.27	ab	25.43±1.46	ab	3.88±0.14	b	0.40±0.01	c	10.74±0.89	cd
		HapB2-6	16.7	4.34±0.18	ab	21.39±0.97	ab	3.55±0.14	ab	0.34±0.01	abc	7.98±0.61	abc
HapB3	3	HapB3-1	22.7	4.36±0.21	a(AB)	21.81±1.15	a(A)	3.45±0.15	a(AB)	0.34±0.02	a(A)	8.17±0.65	a
		HapB3-2*	6.4	3.23±0.53	b(B)	16.37±2.80	b(B)	2.62±0.44	b(B)	0.25±0.04	b(B)	5.38±1.44	a
		HapB3-3	59.9	4.43±0.11	a(A)	21.52±0.58	a(AB)	3.62±0.08	a(A)	0.34±0.01	a(A)	7.97±0.35	a
HapB4	4	HapB4-1	7.9	3.79±0.38	a(A)	18.37±1.73	a(A)	2.97±0.29	a(A)	0.28±0.03	a(A)	6.19±0.92	a(A)
		HapB4-2	14.3	3.90±0.18	a(A)	19.20±0.93	a(A)	3.27±0.15	a(A)	0.32±0.01	a(A)	6.75±0.51	a(A)
		HapB4-3*	30.2	4.53±0.14	ab(AB)	21.42±0.75	a(A)	3.70±0.11	a(A)	0.33±0.01	a(A)	7.85±0.47	a(A)
		HapB4-4	27.4	4.92±0.13	b(B)	24.89±0.70	b(B)	3.83±0.08	b(B)	0.38±0.01	b(B)	9.95±0.42	b(B)
HapB5	3	HapB5-1	58.2	4.40±0.09	a	21.43±0.49	a	3.62±0.07	a	0.34±0.01	a	7.91±0.30	a
		HapB5-2*	19.9	4.50±0.26	a	21.89±1.31	ab	3.36±0.18	a	0.34±0.02	a	8.67±0.76	a
		HapB5-3	17.5	4.92±0.17	a	24.52±0.95	b	3.76±0.12	a	0.36±0.01	a	9.48±0.57	a
HapB6	2	HapB6-1	70.6	4.42±0.09	a(A)	21.64±0.51	a(A)	3.63±0.07	a(A)	0.34±0.01	a(A)	8.00±0.31	a(A)
		HapB6-2*	1.0	1.04±0.03	b(B)	5.31±0.24	b(B)	0.29±0.11	b(B)	0.03±0.01	b(B)	0.15±0.08	b(B)

* Haplotype carried by Sumai 3. Capital and small letters show the significance level at *P*<0.01 and *P*<0.05 compared between haplotypes at the same haplotype block for each trait.

NDS: Number of diseased spikelets; PDS: Percentage of diseased spikelets; LDR: Length of diseased richides; DS: Disease severity; DI: Disease index.

**Table 8:** Genetic effect analyses of Hap combinations between the HapB3 and HapB6.

Hap combination	Number	NDS		PDS		LDR		DS		DI	
		Mean±S.E	*F* test	Mean±S.E	*F* test	Mean±S.E	*F* test	Mean±S.E	*F* test	Mean±S.E	*F* test
HapB3-1/HapB6-1	176	4.40±0.09	a(A)	21.67±0.47	a(A)	3.59±0.06	a(A)	0.34±0.01	a(A)	8.03±0.28	a(A)
HapB3-2/HapB6-1	148	4.33±0.10	a(A)	21.24±0.52	a(A)	3.55±0.07	a(A)	0.34±0.01	a(A)	7.80±0.31	a(A)
HapB3-3/HapB6-1	240	4.42±0.07	a(A)	21.59±0.38	a(A)	3.63±0.05	a(A)	0.34±0.01	a(A)	7.98±0.23	a(A)
HapB3-1/HapB6-2	41	4.20±0.23	a(A)	21.00±1.23	a(AB)	3.30±0.18	a(A)	0.33±0.018	a(A)	7.78±0.67	ab(A)
HapB3-2/HapB6-2	13	2.90±0.50	b(B)	14.67±2.62	b(B)	2.26±0.44	b(B)	0.22±0.04	b(B)	4.57±1.32	b(A)
HapB3-3/HapB6-2	105	4.37±0.12	a(A)	21.21±0.61	a(A)	3.56±0.09	a(A)	0.34±0.01	a(A)	7.82±0.35	a(A)

Capital and small letters show the significance at *P*<0.01 and *P*<0.05 when compairng haplotype combinations for the haplotype block HapB3 and HapB6 for each trait.

NDS: Number of diseased spikelets; PDS: Percentage of diseased spikelets; LDR: Length of diseased richides; DS: Disease severity; DI: Disease index.

### Comparative analysis of genetic and physical maps on 3BS

**Figure 6:**
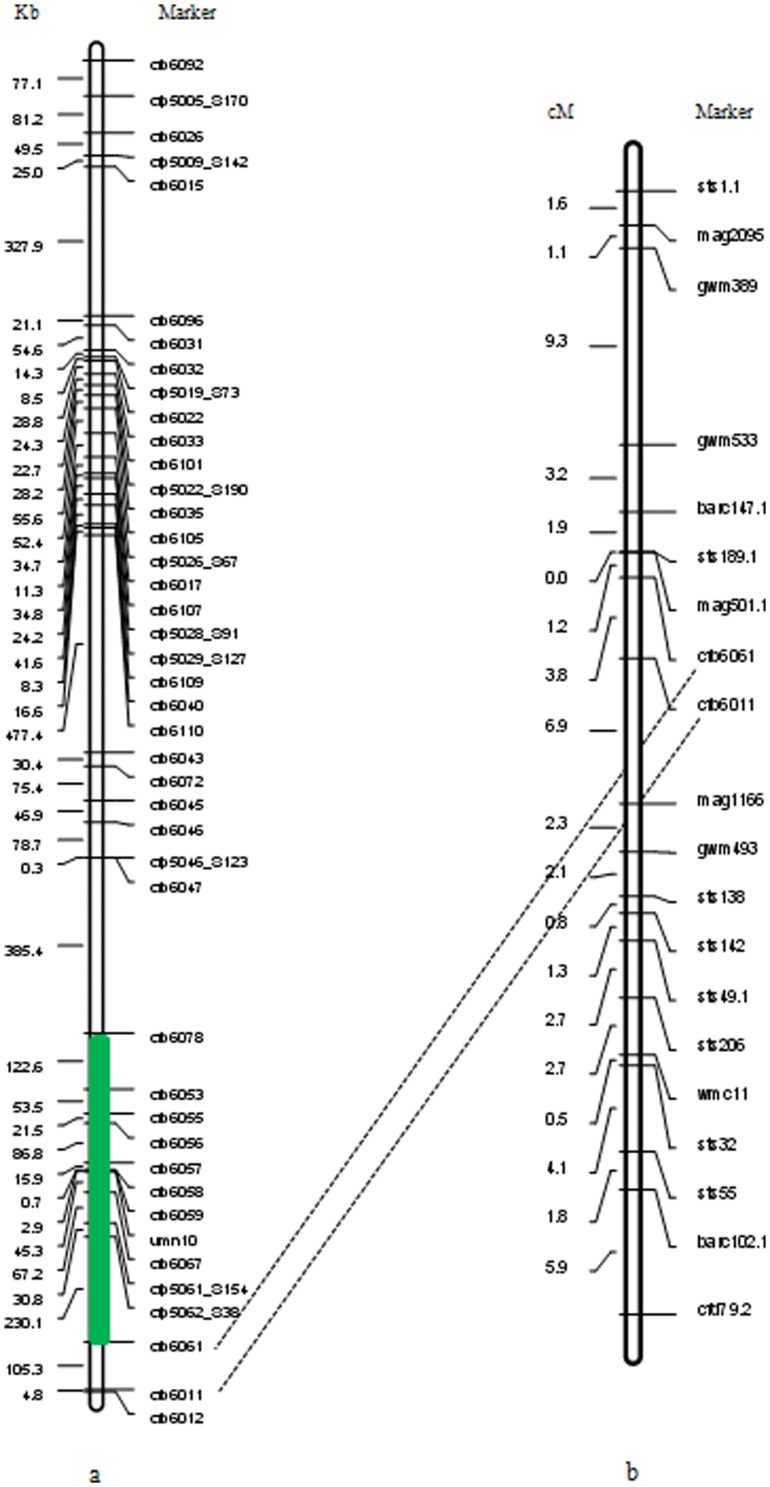
Fine mapping of SSR loci analyzed in the present study. (a) Physical map of molecular markers from sequencing the contig *ctg954* constructed using Chinese Spring. Dashed lines join the same loci on both maps, and the green box in the physical map indicates the region that might include the *Fhb1* (B. Gill pers. comm.). The *umn10* was developed by Liu et al. (2008) [26] as a marker widely used for MAS in FHB-resistance breeding. (b) Genetic map localizing *cfb6061* and *cfb6011* based on a recombinant inbred line (RIL) population (Nanda 2419×Wangshuibai).

Physical map of 42 molecular markers from sequencing the contig *ctg954* (Genebank accession number: FN564434) on the short arm of chromosome 3B was constructed using Chinese Spring wheat (Figure 6a). On the 3.1-Mb genomic region, the interval from 2.2 to 2.8 Mb (between *cfb6078* and *cfb6061*) might include the *Fhb1* locus (B. Gill pers. comm.). Association study of this target region revealed that *cfb6059* was significantly associated with FHB resistance. Importantly, this marker was very close to *umn10* as a marker widely used for MAS in FHB-resistance breeding [26], and theirs physical distance was about 2.9 Kb between them (Figure 6a).

As we all known, *Fhb1* gene was originally located in the distal region of chromosome 3BS between SSR loci *gwm493* and *gwm533* [10]. In order to understand the position parallelism between markers used in our study and others related with FHB resistance, genetic map was constructed based on a recombinant inbred line (RIL) population (Nanda 2419×Wangshuibai) (Figure 6b). Both *cfb6061* and *cfb6011* were mapped the interval between *gwm493* and *gwm533*. Moreover, the physical distance between *cfb6061* and *cfb6059* was 376.3 Kb, while 373.4 Kb between *cfb6061* and *umn10*. Comparative analysis of markers mapped in both genetic and physical maps revealed that *cfb6059* significantly associated with FHB resistance was really located on the region contained *Fhb1* locus, and also very close to the *umn10* marker.

## Discussion

### Efficiency of Association Analysis in the Target Genomic Region

During the past decade, numerous studies have focused on genetic mapping of FHB resistance in wheat, and knowledge on the genetic control of FHB resistance has continually increased with time [16], [17]. Various studies locating QTL in chromosome 3BS are summarized as follows. Using a Sumai 3×Stoa population, the type 2 FHB resistance QTL from Sumai 3 was mapped to chromosome 3BS with the linkage marker *cdo981*, and was designated as *Fhb1* (syn. *Qfhs.ndsu-3BS*) [21]. In one of the first two published QTL mapping studies, Bai et al. [22] found one major QTL in a Ning 7840×Clark population based on AFLP genotyping. Anderson et al. [10] confirmed the major QTL for resistance to fungal spread on chromosome 3BS (*Qfhs.ndsu-3BS*) linked with the marker *gwm493*. This QTL was verified by a series of mapping reports in bi-parental populations. Zhou et al. [23] revealed that the major QTL in a RILs population derived from Ning 7840×Clarkwas also located in the same region on chromosome 3B between SSR markers *gwm533* and *barc147*. At about the same time, Buerstmayr et al. [38] found a major QTL between *gwm533* and *gwm493* on 3BS using a large DH population of CM-82036×Remus. Other intervals or linked markers with the major QTL in this genomic region were *barc133* [39], *gwm533* [11], between *barc133* and *gwm493* [40], between *barc147* and *gwm493* [41], between *gwm533* and *barc147* [42], between *gwm533* and *gwm493* [43], between *sts3B.80* and *sts3B.142* [13], between *sts3B.189* and *sts3B.206* [18] and *umn10* [26]. As already mentioned, this major QTL was distally located on chromosome 3BS between microsatellite markers *gwm493* and *gwm533* [10] (Figure 6b).

In our previous study comparing LD and PIC value levels [33] the intensity of selection along the 3.1-Mb region was variable; for instance, in the interval from 2.2 to 2.8 Mb (between *cfb6078* and *cfb6061*, Figure 6a), which could include the *Fhb1* locus (B. Gill pers. comm.). In the present study, after diversity detection of SSR markers between the two parents, loci *cfb6061* and *cfb6011* were mapped between *gwm533* and *gwm493* using a RIL population of Nanda 2419×Wangshuibai (Figure 6b). Locus *cfb6059* that explained the highest proportion of phenotypic variance (6.2%) was between *cfb6078* and *cfb6061*, and very close (261 Kb) to the mapped marker *cfb6061*. Comparing the physical map of SSR markers constructed using Chinese Spring (Figure 6a) with the genetic map (Figure 6b), all associated markers detected in this study were located in the genomic region indicated by numerous linkage studies.

In addition, this significantly associated marker *cfb6059* was very close to *umn10* as a marker widely used for MAS [26], and theirs physical distance was about 2.9 Kb between them (Figure 6a), which indicates a complementation relationship between association study and linkage analysis using different mapping populations. To further dissect the relatedness of these two markers, we genotyped all our materials using the marker *umn10* designed by Liu et al. [26]. All accessions contained two types of alleles at this locus, i.e. 236 bp (susceptible control) and 239 bp (resistant control), and *F*-test based on our natural populations found that none of significant associations between *unm10* and FHB-related traits existed in both the European and Asian subgroups (Table S4), but five of six resistant germplasm resources (Figure S1, Table S3), except Liaochun 4 (Liaoning, China), had the same allele 239 bp at this locus with the famous resistant resource Sumai 3, indicating it was an effective diagnostic marker for MAS for gene *Fhb1*. Moreover, the linkage disequilibrium (LD) between *cfb6059* and *umn10* was *r^2^* = 0.28 (*P*<0.001) in despite of their closely linkage, revealing that there was a complementary relationship between these two markers in marker-assisted selection of FHB-resistant breeding.

### Complementary Relatedness between Markers and Haplotype Association Analysis

Association mapping based on linkage disequilibrium (LD), representing next-generation plant genetics [44], [45], has become a powerful tool for dissecting complex agronomic traits and identifying specific alleles conferring target traits using natural crop populations [28], [34], [35], [46]. Generally, population structure and genetic relatedness influence the success of association analysis, but may result in spurious marker-trait associations [47]. One effective strategy to address this problem is the compressed mixed linear model (MLM) suggested by Yu et al. [36] and Zhang et al. [37] based on the chosen Q-matrix derived from STRUCTURE and the kinship-matrix from SPAGeDi. A haplotype was described as a set of closely linked intra-chromosome molecular markers that tend to be inherited together [32]. Haplotype association is likely to be more powerful in the presence of LD [31] and haplotype-trait association analyses are helpful for precise mapping of important genomic regions and location of superior or preferred alleles or haplotypes for breeding [48]. Recently, there has been an emphasis on haplotype (or haplotype block) analysis in many crops. For example, Malysheva-Otto and Röder [49] discovered novel haplotypes and analyzed their distribution and significance in the endosperm-specific *β*-amylase gene *Bmy1* of cultivated barley (*Hordeum vulgare* L.); Stracke et al. [50] showed that the linkage disequilibrium pattern and haplotype structure led to interplay between low recombination and recent breeding history surrounding a locus encoding *Bymovirus* resistance in barley; and Gore et al. [51] generated a first-generation haplotype map of maize to provide a foundation for uniting breeding efforts and for dissecting complex traits through genome-wide association studies.

With the increasing number of studies on association mapping using grouped molecular markers or several haplotypes, the efficiency of the two methodologies has greatly improved. In the present study, both marker association (Figure 3, Table 4) and haplotype association (Figure 5, Tables 6, 7) were conducted for five FHB-related traits in a 3.1-Mb genomic region on wheat chromosome 3BS. Four markers and two haplotypes were significantly associated with FHB resistance. These four markers were located within or surrounding two haplotype blocks (Figure 5); HapB3 and HapB6 each contained one haplotype significantly associated with FHB-related traits (Table 7). Further study revealed that these two haplotypes had strong interactive effects on FHB response (Table 8). Thus there was a good complementary relatedness between molecular markers and haplotype associations in the target chromosome region. As shown in Table S3, except Youzimai (Sichuan, China), other five materials contained not only similar disease rating but also same haplotype comparing with Sumai 3.

### Molecular Markers and Haplotypes Associated with FHB-related Traits

Using compressed MLM, association analysis between five FHB-related traits and 42 molecular markers along the 3.1-Mb region detected 12 significant association signals at the threshold of *P*<0.05 (Figure 3, Table 4), and locus *cfb6059* explained the highest proportion of variation (6.2%) for the trait NDS mapped in the interval between *gwm493* and *gwm533* (Figure 6a, b), indicating a good integration of association mapping and QTL mapping in the discovery of molecular markers associated with *Fhb1*. Association analysis also validates superior or preferred alleles in germplasm collections [52]. For example, Zhang et al. [44] found that allele *Xgwm130_132_* underwent very strong positive selection for 1,000 kernel weight during modern breeding. Through association mapping of dynamic development of plant height in common wheat, Zhang et al. [53] exposed different allelic effects of associated markers; *gwm495-4B_155_* was associated with reduced height of −11.2 cm under drought stressed and −15.3 cm under well watered conditions, whereas the *167 bp* allele exhibited increased height effects of 3.9 and 8.1 cm, respectively. In the present study, different allelic effects of significant loci were also found for FHB response (Figure 4, Figure S3). Wheat accessions such as Sumai 3 with a *289* bp allele at *cfb6110* had lower mean phenotypic values for NDS, PDS, LDR and DI than those carrying a *294* bp allele. Taking PDS as a trait example (Figure 4), negative genetic effects were detected for the *236* and *238* bp alleles, but there were positive effects when the allele *242 bp* was present. Thus the identification of superior alleles will help in choosing parents for crossing programs, to ensure maximum numbers of superior alleles across sets of loci targeted for selection. Fixation of those alleles can then be targeted [54].

As suggested by Garner and Slatkin [31], the presence of LD is the premise of haplotype association for precise mapping of important genomic regions. In this study, the identification of haplotype blocks was performed by a sliding window LD of 5 markers as the LD window size at the level of *r^2^*>0.1 and *P*<0.001 between random closely linked markers. Six HapBs were detected in the 3.1-Mb genomic region (Figure 5, Table 6). HapB3-2 and HapB6-2 conferred the lowest phenotypic values in five FHB-related traits. In addition, two landmark FHB resistance germplasms, Sumai 3 and Wangshuibai, carry these two sub-haplotype blocks. Interestingly, HapB6 included the significantly associated loci *cfb6059* and *cfp5062_S38*, suggesting that the *Fhb1* locus might be included in this ∼392 Kb haplotype block. Based on analyses of genes identified in the sequenced contig *Ctg954* of wheat chromosome 3B [55], the 392 Kb region was the interval that included the highest number of genes, and marker *cfb6059* was located between genes *ctg0954b.00390.1* and *ctg0954b.00400.1* (F. Choulet pers. comm.). On the other hand, HapB3 did not involve any associated locus, suggesting a complementary relationship between haplotype analysis and simple association mapping. The locus *cfb6110* significantly associated with scab-related traits was very close physically to haplotype block HapB3. It can be predicted that *Fhb1* should be within the HapB6, but we cannot exclude the possibility of another gene located around HapB3. This study therefore provides useful information and genetic markers for cloning *Fhb1* and for marker-assisted selection in breeding.

## Materials and Methods

### Plant Materials

Two hundred and sixty six wheat accessions comprising 157 Asian and 91 European genotypes were used in this study. They were chosen according to their heading date in China from those included in the previous study on genetic diversity and linkage disequilibrium in 376 Asian and European bread wheat accessions [33]. In addition, four lines, Sumai 3 (Jiangsu) and Wangshuibai (Jiangsu), and Ningmai 11 (Jiangsu) and Mianyang 11 (Sichuan) were used as resistant and susceptible controls in phenotyping of FHB response, respectively. Their detailed information including pedigree data is given in Table S5.

### Fusarium Head Blight Response Assays

FHB responses were assessed in three environments, viz. Jiangpu Experimental Station, Nanjing Agricultural University in 2009 (NAU_2009, E1), experimental field of Jiangsu Academy of Agricultural Sciences in 2009 (JAAS_2009, E2) and Jiangpu Experimental Station, NAU in 2010 (NAU_2010, E3). Each accession was planted in two replicates with fifteen seeds evenly distributed in a 1.5 m row, with 25 cm between rows.

All accessions were evaluated for FHB response based on the single floret inoculation method. A mixed *F. graminearum* conidial suspension containing four virulent strains with a concentration of 5×10^4^ conidial/ml was produced as described by [15], [20]. Inoculation was carried out at anthesis with 20 ul of conidial suspension inserted into the floral cavity between the lemma and palea of a single floret in the middle of a spike. Inoculated spikes were humidified in an enclosed plastic chamber for 2 days to induce infection. Ten plants were selected from each row for evaluation 21 days after inoculation. Number of diseased spikelets (NDS) and the length of diseased rachides (LDR) for each inoculated plant, and its corresponding spikelet number per spike (SPN) spike length (SPL) were investigated. Three phenotypic parameters were calculated according to the formula: PDS (percentage of diseased spikelets) = (NDS/SPN)×100%, DS (disease severity) = LDR/SPL, and DI (disease index) = PDS×DS. The final phenotypic data included five FHB response traits, viz. NDS, LDR (cm), PDS (%), DS and DI (%) evaluated in three environments. The mean values are listed in Table S6. Statistical calculations of correlations between environments and between traits were performed with SPSS v12.0.

### Genotyping, Population Structure, and Linkage Disequilibrium

In the previous study, Hao et al. [33] used 42 molecular markers (32 SSR plus 10 SNP) from the contig *ctg954* (Genebank accession number: FN564434) [55], [56] (Table S7) and 70 SSR markers from the whole genome to genotype 376 wheat accessions including the 266 accessions selected for the current study. The four controls were genotyped using the 42 3B-specific markers based on the method described in Hao et al. [33], and the marker *umn10* designed by Liu et al. [26] was also used to genotype all materials. Then, two sets of genotyping data in both the 3.1- Mb sequenced region and the whole genome were used in this study (Table S8). Additionally, a recombinant inbred line (RIL) population (Nanda 2419×Wangshuibai) was genotyped using 32 SSRs to make fine mapping of these markers in the genomic region.

Additional analyses included population structure (Q value) with STRUCTURE v2.2 software [57], estimation of the most appropriate number of sub-groups (Δk value) on the basis of the Evanno et al. [58] criterion using 70 genome-wide SSR, squared allele-frequency correlations (*r^2^*) and significances of each pair of loci using the 32 polymorphic SSRs and 10 SNPs in the approximate 3.1-Mb region on chromosome 3BS with the dedicated procedure of the TASSEL v3.0 software [37]. All parameter settings for population structure analysis and linkage disequilibrium evaluation in the present study were based on the method of Hao et al. [33], [59]. In addition, we also produced a hierarchical clustering tree from the Manhattan dissimilarity matrix using DARwin v5 software [60] based on five phenotypic traits, and principal coordinate analysis of the test material to reveal relationships among accessions using NTSYS-pc version2.1 software [61].

In order to define the degree of genetic covariance between pairs of individuals, the relative kinship matrix (K) was calculated using genotypic data of genome-wide SSR with SPAGeDi software [62]. Calculation of pairwise kinship coefficients was according to Loiselle et al. [63] with 10,000 permutation tests. Negative values between individual pairs were changed to 0, as this indicated a lower than expected relationship between two random individuals [36].

### Marker-trait Association

The software program TASSEL v3.0 (http://www2.maizegenetics.net/) [37], [64] was used to calculate associations between the 42 3B-specific markers and phenotypic traits of scab resistance, with the compressed mixed linear model (MLM) suggested by Yu et al. [36] and Zhang et al. [37] based on the chosen Q-matrix derived from STRUCTURE and the kinship-matrix from SPAGeDi. For estimating associations those markers with the allelic frequencies less than 0.05 were filtered as rare alleles and deleted. We adopted the MLM options of optimum level and population parameters previously determined (P3D). The significance levels (*P* values) between markers and traits and phenotypic variation (*R^2^*) explained by the markers associated with traits were obtained for further study. Markers were defined as being significantly associated with traits according to their *P* values (-Log*P*>1.30, *P*<0.05).

Based on the procedure of the TASSEL software, only using phenotypic data and the kinship matrix, the heritability (*h^2^*) of each test trait in different environments, defined as the proportion of genetic variance over the total variance, was calculated according to the formula *h^2^* = σ_a_^2^/(σ_a_^2^+σ_e_^2^) with the MLM options of no compression and re-estimation for each marker. Here, σ_a_^2^ means genetic variance, and σ_e_^2^ indicates the residual variance. Allelic effects were evaluated in comparison to the “null allele” (missing plus rare alleles) for each locus [52].

### Haplotype Mapping

To determine the number and types of haplotype blocks (HapB) along the contig *ctg954*, the sliding window LD, with 5 markers as LD window size, was managed through TASSEL v3.0 [37], [64], and the markers were filtered for rare alleles with frequencies of less than 5% in the whole collection. For assignment of one HapB, *r^2^*>0.1 and *P*<0.001 between random closely linked markers were regarded as the threshold in the sliding window LD. Based on different allelic combinations of all loci in the same HapB, the haplotype (Hap) was statistically calculated for each HapB in Excel.

## Supporting Information

Figure S1 **Hierarchical clustering (UPGMA) of screened accessions based on a Manhattan dissimilarity matrix using five FHB-related traits.** Blue line means negative control, and red line indicates positive control.
(DOC)

Figure S2 **Association studies of five FHB-related traits with molecular markers in the 3.1-Mb genomic region.** (a) Dot plots of compressed mixed linear model (MLM) for percentage of diseased spikelets (PDS). Negative log_10_-transformed *P* values in a sequenced contig (*ctg954*) of 3.1-Mb are plotted against position along the contig. Blue horizontal dashed line indicates the chromosome-region significance threshold. (b) Quantile-quantile plot of compressed MLM for PDS. (c) Dot plots of compressed MLM for disease severity (DS), as in a. (d) Quantile-quantile plot of compressed MLM for DS. (e) Dot plots of compressed MLM for disease index (DI), as in a. (f) Quantile-quantile plot of compressed MLM for DI.
(DOC)

Figure S3 **Comparison of allelic effects of four loci significantly associated with FHB-related traits NDS (a), LDR (b), DS (c) and DI (d).**
(DOC)

Table S1 FHB-related traits of controls in different environments.
(DOC)

Table S2 Correlation analyses of five FHB-related traits.
(DOC)

Table S3 FHB-related traits of wheat accessions clustered into the same subgroup as Sumai 3 by UPGMA based on a Manhattan dissimilarity matrix.
(DOC)

Table S4 Comparison of FHB-related traits between the two alleles at *umn10* in both European and Asian wheat gene pools.
(DOC)

Table S5 Passport data of 266 wheat accessions with detailed pedigree information and four controls included in the study.
(XLS)

Table S6 FHB resistance evaluation data in three environments and theirs mean value.
(XLS)

Table S7 The sequences of molecular markers and theirs physical positions on *ctg954* BACs contig.
(XLS)

Table S8 Genotyping data of all accessions for 43 3B-specific markers and 70 whole-genome SSR markers.
(XLS)

## References

[1] Gilbert J, Tekauz A (2000) Review: recent developments in research on fusarium head blight of wheat in Canada. Can J Plant Pathol 22: 1–8.

[2] Bai GH, Shaner G (1994) Scab of wheat: prospects for control. Plant Disease 78: 760–766.

[3] Dexter JE, Clear RM, Preston KR (1996) Fusarium head blight: effect on the milling and baking of some Canadian wheats. Cereal Chem 73: 695–701.

[4] Dexter JE, Marchylo B, Clear RM, Clarke JM (1997) Effect of fusarium head blight on semolina milling and pasta-making quality of durum wheat. Cereal Chem 74: 519–525.

[5] McMullen M, Jones R, Gallenberg D (1997) Scab of wheat and barley: a re-emerging disease of devastating impact. Plant Dis 81: 1340–1348. 10.1094/PDIS.1997.81.12.1340 30861784

[6] Snijders CHA (1994) Breeding for resistance to fusarium in wheat and maize. In: Miller JD, Trenholm HL (eds) Mycotoxins in grain: compounds other than aflatoxin. St. Paul, MN: Eagan. pp. 37–58.

[7] Liu S, Anderson JA (2003) Marker assisted evaluation of *Fusarium* head blight resistant wheat germplasm. Crop Sci 43: 760–766.

[8] Yu BY, Bai GH, Cai SB, Dong YH, Ban T (2008) New *Fusarium* head blight-resistant sources from Asian wheat germplasm. Crop Sci 48: 1090–1097.

[9] McCartney CA, Somers DJ, Fedak G, Cao W (2004) Haplotype diversity at fusarium head blight resistance QTLs in wheat. Theor Appl Genet 109: 261–271. 15057418 10.1007/s00122-004-1640-x

[10] Anderson JA, Stack RW, Liu S, Waldron BL, Fjeld AD, et al (2001) DNA markers for Fusarium head blight resistance QTLs in two wheat populations. Theor Appl Genet 102: 1164–1168.

[11] Somers DJ, Fedak G, Savard M (2003) Molecular mapping of novel genes controlling *Fusarium* head blight resistance and deoxynivalenol accumulation in spring wheat. Genome 46: 555–564. 12897863 10.1139/g03-033

[12] Steiner B, Lemmens M, Griesser M, Scholz U, Schondelmaier J, et al (2004) Molecular mapping of resistance to *Fusarium* head blight in the spring wheat cultivar Frontana. Theor Appl Genet 109: 215–224. 14997302 10.1007/s00122-004-1620-1

[13] Cuthbert PA, Somers DJ, Thomas J, Cloutier S, Brulé-Babel A (2006) Fine mapping *Fhb1*, a major gene controlling fusarium head blight resistance in bread wheat (*Triticum aestivum* L.). Theor Appl Genet 112: 1465–1472. 16518614 10.1007/s00122-006-0249-7

[14] Ma ZQ, Xue SL, Lin F, Yang SH, Li GQ, et al (2008) Mapping and validation of scab resistance QTLs in the Nanda2419×Wangshuibai population. Cereal Res Commun (Supplementum B) 36: 245–251.

[15] Xue SL, Xu F, Tang MZ, Zhou Y, Li GQ, et al (2011) Precise mapping *Fhb5*, a major QTL conditioning resistance to *Fusarium* infection in bread wheat (*Triticum aestivum* L.). Theor Appl Genet 123: 1055–1063. 21739138 10.1007/s00122-011-1647-z

[16] Buerstmayr H, Ban T, Anderson JA (2009) QTL mapping and marker-assisted selection for *Fusarium* head blight resistance in wheat: a review. Plant Breeding 128: 1–26.

[17] Löffler M, Schön CC, Miedaner T (2009) Revealing the genetic architecture of FHB resistance in hexaploid wheat (*Triticum aestivum* L.) by QTL meta-analysis. Mol Breed 23: 473–488.

[18] Liu S, Zhang X, Pumphrey MO, Stack RW, Gill BS, et al (2006) Complex microcolinearity among wheat, rice and barley revealed by fine mapping of the genomic region harboring a major QTL for resistance to *Fusarium* head blight in wheat. Funct Integr Genomics 6: 83–89. 16270217 10.1007/s10142-005-0007-y

[19] Cuthbert PA, Somers DJ, Brulé-Babel A (2007) Mapping of *Fhb2* on chromosome 6BS: a gene controlling Fusarium head blight field resistance in bread wheat (*Triticum aestivum* L.). Theor Appl Genet 114: 429–437. 17091262 10.1007/s00122-006-0439-3

[20] Xue SL, Li GQ, Jia HY, Xu F, Lin F, et al (2010) Fine mapping *Fhb4* a major QTL conditioning resistance to Fusarium infection in bread wheat (*Triticum aestivum* L.). Theor Appl Genet 121: 147–156. 20198469 10.1007/s00122-010-1298-5

[21] Waldron BL, Moreno-Sevilla B, Anderson JA, Stack RW, Frohberg RC (1999) RFLP mapping of QTL for Fusarium head blight resistance in wheat. Crop Sci 39: 805–811.

[22] Bai G, Kolb FL, Shaner G, Domier LL (1999) Amplified fragment length polymorphism markers linked to a major quantitative trait locus controlling scab resistance in wheat. Phytopathology 89: 343–348. 18944781 10.1094/PHYTO.1999.89.4.343

[23] Zhou WC, Kolb FL, Bai G, Shaner G, Domier LL (2002) Genetic analysis of scab resistance QTL in wheat with microsatellite and AFLP markers. Genome 45: 719–727. 12175075 10.1139/g02-034

[24] Yang ZP, Gilbert J, Fedak G, Somers DJ (2005) Genetic characterization of QTL associated with resistance to *Fusarium* head blight in a doubled-haploid spring wheat population. Genome 48: 187–196. 15838540 10.1139/g04-104

[25] Pumphrey MO, Bernardo R, Anderson JA (2007) Validating the *Fhb1* QTL for *fusarium* head blight resistance in near isogenic wheat lines developed from breeding populations. Crop Sci 47: 200–206.

[26] Liu S, Pumphrey MO, Gill BS, Trick HN, Zhang JX, et al (2008) Toward positional cloning of *FHB1*, a major QTL for fusarium head blight resistance in wheat. Cereal Res Commun (Suppl B) 36: 195–201.

[27] Flint-Garcia SA, Thornsberry JM, Buckler ES (2003) Structure of linkage disequilibrium in plants. Annu Rev Plant Biol 54: 357–374. 14502995 10.1146/annurev.arplant.54.031902.134907

[28] Atwell S, Huang YS, Vilhjálmsson BJ, Willems G, Horton M, et al (2010) Genome-wide association study of 107 phenotypes in *Arabidopsis thaliana* inbred lines. Nature 465: 627–631. 20336072 10.1038/nature08800 PMC3023908

[29] Zwart RS, Muylle H, Van Bockstaele E, Roldán-Ruiz I (2008) Evaluation of genetic diversity of *Fusarium* head blight resistance in European winter wheat. Theor Appl Genet 117: 813–828. 18587558 10.1007/s00122-008-0822-3

[30] Miedaner T, Würschum T, Maurer HP, Korzun V, Ebmeyer E, et al (2011) Association mapping for *Fusarium* head blight resistance in European soft winter wheat. Mol Breed 28: 647–655.

[31] Garner C, Slatkin M (2003) On selecting markers for association studies: patterns of linkage disequilibrium between two and three diallelic loci. Genet Epidemiol 24: 57–67. 12508256 10.1002/gepi.10217

[32] Andersen J, Lübberstedt T (2003) Functional markers in plants. Trends Plant Sci 8: 554–560. 14607101 10.1016/j.tplants.2003.09.010

[33] Hao CY, Perretant MR, Choulet F, Wang LF, Paux E, et al (2010) Genetic diversity and linkage disequilibrium studies on a 3.1-Mb genomic region of chromosome 3B in European and Asian bread wheat (*Triticum aestivum* L.) populations. Theor Appl Genet 121: 1209–1225. 20559816 10.1007/s00122-010-1382-x

[34] Yu J, Buckler E (2006) Genetic association mapping and genome organization of maize. Curr Opin Biotech 17: 155–160. 16504497 10.1016/j.copbio.2006.02.003

[35] Yan JB, Shah T, Warburton ML, Buckler ES, McMullen MD, et al (2009) Genetic characterization and linkage disequilibrium estimation of a global maize collection using SNP markers. Plos One 4 12:e8451 doi:10.1371/journal.pone.0008451. 20041112 10.1371/journal.pone.0008451 PMC2795174

[36] Yu JM, Pressoir G, Briggs WH, Bi IV, Yamasaki M, et al (2006) A unified mixed-model method for association mapping that accounts for multiple levels of relatedness. Nat Genet 38: 203–208. 16380716 10.1038/ng1702

[37] Zhang ZW, Ersoz E, Lai CQ, Fodhunter RJ, Tiwari HK, et al (2010) Mixed linear model approach adapted for genome-wide association studies. Nat Genet 42: 355–360. 20208535 10.1038/ng.546 PMC2931336

[38] Buerstmayr H, Lemmens M, Hartl L, Doldi L, Steiner B, et al (2002) Molecular mapping of QTL for *Fusarium* head blight resistance in spring wheat. I. Resistance to fungal spread (type II resistance). Theor Appl Genet 104: 84–91. 12579431 10.1007/s001220200009

[39] Bourdoncle W, Ohm HW (2003) Quantitative trait loci for resistance to Fusarium head blight in recombinant inbred wheat lines from the cross Huapei 57-2/Patterson. Euphytica 131: 131–136.

[40] Shen X, Zhou M, Lu W, Ohm H (2003) Detection of Fusarium head blight resistance QTL in a wheat population using bulked segregant analysis. Theor Appl Genet 106: 1041–1047. 12671752 10.1007/s00122-002-1133-8

[41] Zhang X, Zhou MP, Ren LJ, Bai GH, Ma HX, et al (2004) Molecular characterization of Fusarium head blight resistance from wheat variety Wangshuibai. Euphytica 139: 59–64.

[42] Lin F, Kong ZX, Zhu HL, Xue SL, Wu JZ, et al (2004) Mapping QTL associated with resistance to *Fusarium* head blight in the Nanda2419×Wangshuibai population. I. Type II resistance. Theor Appl Genet 109: 1504–1511. 15290053 10.1007/s00122-004-1772-z

[43] Lemmens M, Scholz U, Berthiller F, Dall'Asta C, Koutnik A, et al (2005) The ability to detoxify the mycotoxin deoxynivalenol colocalizes with a major quantitative trait locus for fusarium head blight resistance in wheat. Mol Plant Microbe Interact 18: 1318–1324. 16478051 10.1094/MPMI-18-1318

[44] Zhang XY, Tong YP, You GX, Hao CY, Ge HM, et al (2007) Hitchhiking effect mapping: A new approach for discovering agronomically important genes. Agri Sci China 6: 255–264.

[45] Nordborg M, Weigel D (2008) Next-generation genetics in plants. Nature 456: 720–723. 19079047 10.1038/nature07629

[46] Yan JB, Warburton M, Crouch J (2011) Association mapping for enhancing maize (*Zea mays* L.) genetic improvement. Crop Sci 51: 433–449.

[47] Gupta P, Rustgi S, Kulwal P (2005) Linkage disequilibrium and association studies in higher plants: present status and future prospects. Plant Mol Biol 57: 461–485. 15821975 10.1007/s11103-005-0257-z

[48] Barrero RA, Bellgard M, Zhang X (2011) Diverse approaches to achieving grain yield in wheat. Funct Integr Genomics 11: 37–48. 21221697 10.1007/s10142-010-0208-x

[49] Malysheva-Otto LV, Röder MS (2006) Haplotype diversity in the endosperm specific *β*-amylase gene *Bmy1* of cultivated barley (*Hordeum vulgare* L.). Mol Breeding 18: 143–156.

[50] Stracke S, Presterl T, Stein N, Perovic D, Ordon F, et al (2007) Effects of introgression and recombination on haplotype structure and linkage disequilibrium surrounding a locus encoding *Bymovirus* resistance in barley. Genetics 175: 805–817. 17151251 10.1534/genetics.106.063800 PMC1800611

[51] Gore MA, Chia JM, Elshire RJ, Sun Q, Ersoz ES, et al (2009) A first-generation haplotype map of maize. Science 326: 1115–1117. 19965431 10.1126/science.1177837

[52] Breseghello F, Sorrells ME (2006) Association mapping of kernel size and milling quality in wheat (*Triticum aestivum* L.) cultivars. Genetics 172: 1165–1177. 16079235 10.1534/genetics.105.044586 PMC1456215

[53] Zhang JN, Hao CY, Ren Q, Chang XP, Liu GR, et al (2011) Association mapping of dynamic developmental plant height in common wheat. Planta 234: 891–902. 21647605 10.1007/s00425-011-1434-8

[54] Koebner RMD, Summers RW (2003) 21st century wheat breeding: plot selection or plate detection. Trends in Biotechnology 21: 59–63. 12573853 10.1016/S0167-7799(02)00036-7

[55] Choulet F, Wicker T, Rustenholz C, Paux E, Salse J, et al (2010) Megabase level sequencing reveals contrasted organization and evolution patterns of the wheat gene and transposable element spaces. Plant Cell 22: 1686–1701. 20581307 10.1105/tpc.110.074187 PMC2910976

[56] Paux E, Faure S, Choulet F, Roger D, Gauthier V, et al (2010) Insertion site-based polymorphism markers open new perspectives for genome saturation and marker-assisted selection in wheat. Plant Biotechnol J 8: 196–210. 20078842 10.1111/j.1467-7652.2009.00477.x

[57] Pritchard JK, Stephens M, Donnelly P (2000) Inference of population structure using multilocus genotype data. Genetics 155: 945–959. 10835412 10.1093/genetics/155.2.945 PMC1461096

[58] Evanno G, Regnaut S, Goudet J (2005) Detecting the number of clusters of individuals using the software STRUCTURE: a simulation study. Mol Ecol 14: 2611–2620. 15969739 10.1111/j.1365-294X.2005.02553.x

[59] Hao CY, Wang LF, Ge HM, Dong YC, Zhang XY (2011) Genetic diversity and linkage disequilibrium in Chinese bread wheat (*Triticum aestivum* L.) revealed by SSR markers. Plos One 6 2:e17279 doi: 10.1371/journal.pone.0017279. 21365016 10.1371/journal.pone.0017279 PMC3041829

[60] Perrier X, Flori A, Bonnot F (2003) Data analysis methods. In: Hamon P, Seguin M, Perrier X, Glaszmann JC (eds) Genetic diversity of cultivated tropical plants. Enfield, Science Publishers, Montpellier, pp 43–76.

[61] Rohlf FJ (2000) NTSYS-pc: numerical taxonomy and multivariate analysis system, version 2.1. Exeter Software, Setauket, NY.

[62] Hardy OJ, Vekemans X (2002) SPAGeDi: a versatile computer program to analyze spatial genetic structure at the individual or population levels. Mol Ecol Notes 2: 618–620.

[63] Loiselle BA, Sork VL, Nason J, Graham C (1995) Spatial genetic structure of a tropical understory shrub, Psychotria officinalis (Rubiaceae). Am J Bot 82: 1420–1425.

[64] Bradbury PJ, Zhang ZW, Kroon DE, Casstevens TM, Ramdoss Y, et al (2007) TASSEL: software for association mapping of complex traits in diverse samples. Bioinformatics 23: 2633–2635. 17586829 10.1093/bioinformatics/btm308

