# Supplementary material for: Association Mapping and Haplotype Analysis of a 3.1-Mb Genomic Region Involved in Fusarium Head Blight Resistance on Wheat Chromosome 3BS
Source: PLoS One. 2012 Oct 5;7(10):e46444. doi: 10.1371/journal.pone.0046444 (PMC3465345; doi:10.1371/journal.pone.0046444)
Supplement: Figure S1 — Hierarchical clustering (UPGMA) of screened accessions based on a Manhattan dissimilarity matrix using five FHB-related traits. Blue line means negative control, and red line indicates positive control. (DOC) [file pone.0046444.s001.doc]

**Figure S1. Hierarchical clustering (UPGMA) of screened accessions based on a Manhattan dissimilarity matrix using five FHB-related traits.** Blue line means negative control, and red line indicates positive control.
