# Supplementary material for: Association Mapping and Haplotype Analysis of a 3.1-Mb Genomic Region Involved in Fusarium Head Blight Resistance on Wheat Chromosome 3BS
Source: PLoS One. 2012 Oct 5;7(10):e46444. doi: 10.1371/journal.pone.0046444 (PMC3465345; doi:10.1371/journal.pone.0046444)
Supplement: Figure S2 — Association studies of five FHB-related traits with molecular markers in the 3.1-Mb genomic region. (a) Dot plots of compressed mixed linear model (MLM) for percentage of diseased spikelets (PDS). Negative log10-transformed P values in a sequenced contig (ctg954) of 3.1-Mb are plotted against position along the contig. Blue horizontal dashed line indicates the chromosome-region significance threshold. (b) Quantile-quantile plot of compressed MLM for PDS. (c) Dot plots of compressed MLM for disease severity (DS), as in a. (d) Quantile-quantile plot of compressed MLM for DS. (e) Dot plots of compressed MLM for disease index (DI), as in a. (f) Quantile-quantile plot of compressed MLM for DI. (DOC) [file pone.0046444.s002.doc]

**Figure S2. Association studies of five FHB-related traits with molecular markers in the 3.1-Mb genomic region.** (a) Dot plots of compressed mixed linear model (MLM) for percentage of diseased spikelets (PDS). Negative log_10_-transformed *P* values in a sequenced contig (*ctg954*) of 3.1-Mb are plotted against position along the contig. Blue horizontal dashed line indicates the chromosome-region significance threshold. (b) Quantile-quantile plot of compressed MLM for PDS. (c) Dot plots of compressed MLM for disease severity (DS), as in a. (d) Quantile-quantile plot of compressed MLM for DS. (e) Dot plots of compressed MLM for disease index (DI), as in a. (f) Quantile-quantile plot of compressed MLM for DI.
