# Supplementary material for: Association Mapping and Haplotype Analysis of a 3.1-Mb Genomic Region Involved in Fusarium Head Blight Resistance on Wheat Chromosome 3BS
Source: PLoS One. 2012 Oct 5;7(10):e46444. doi: 10.1371/journal.pone.0046444 (PMC3465345; doi:10.1371/journal.pone.0046444)
Supplement: Figure S3 — Comparison of allelic effects of four loci significantly associated with FHB-related traits NDS (a), LDR (b), DS (c) and DI (d). (DOC) [file pone.0046444.s003.doc]

**Figure S3. Comparison of allelic effects of four loci significantly associated with FHB-related traits NDS (a), LDR (b), DS (c) and DI (d).**
