# Supplementary material for: Association Mapping and Haplotype Analysis of a 3.1-Mb Genomic Region Involved in Fusarium Head Blight Resistance on Wheat Chromosome 3BS
Source: PLoS One. 2012 Oct 5;7(10):e46444. doi: 10.1371/journal.pone.0046444 (PMC3465345; doi:10.1371/journal.pone.0046444)
Supplement: Table S1 — FHB-related traits of controls in different environments. (DOC) [file pone.0046444.s004.doc]

**Table S1 FHB-related traits of controls in different environments.**

| Trait | E1 | | E2 | | E3 | | | | T | | | |
| --- | --- | --- | --- | --- | --- | --- | --- | --- | --- | --- | --- | --- |
|  | CK3 | CK4 | CK1 | CK2 | CK1 | CK2 | CK3 | CK4 | CK1 | CK2 | CK3 | CK4 |
| NDS | 1.00 | 2.85 | 1.10 | 4.62 | 1.04 | 4.20 | 1.03 | 3.85 | 1.07 | 4.41 | 1.01 | 3.35 |
| PDS | 5.09 | 14.68 | 5.80 | 22.59 | 5.30 | 19.51 | 5.06 | 20.34 | 5.55 | 21.05 | 5.07 | 17.51 |
| LDR | 0.11 | 3.27 | 0.41 | 4.17 | 0.39 | 3.38 | 0.24 | 3.22 | 0.40 | 3.78 | 0.18 | 3.25 |
| DS | 0.01 | 0.32 | 0.04 | 0.37 | 0.04 | 0.35 | 0.02 | 0.38 | 0.04 | 0.36 | 0.01 | 0.35 |
| DI | 0.05 | 4.71 | 0.24 | 8.26 | 0.22 | 6.73 | 0.10 | 7.72 | 0.23 | 7.50 | 0.07 | 6.22 |

Note: CK1: Sumai 3; CK2: Ningmai 11; CK3: Wangshuibai; CK4: Mianyang 11.

T: total; E1, E2, E3 are different environments as in Table 1.

NDS: Number of diseased spikelets; PDS: Percentage of diseased spikelets; LDR: Length of diseased richides; DS: Disease severity; DI: Disease index.
