# Supplementary material for: Association Mapping and Haplotype Analysis of a 3.1-Mb Genomic Region Involved in Fusarium Head Blight Resistance on Wheat Chromosome 3BS
Source: PLoS One. 2012 Oct 5;7(10):e46444. doi: 10.1371/journal.pone.0046444 (PMC3465345; doi:10.1371/journal.pone.0046444)
Supplement: Table S2 — Correlation analyses of five FHB-related traits. (DOC) [file pone.0046444.s005.doc]

**Table S2 Correlation analyses of five FHB-related traits.**

|  | NDS | PDS | LDR | DS | DI |
| --- | --- | --- | --- | --- | --- |
| NDS | 1 | 0.950** | 0.843** | 0.859** | 0.916** |
| PDS |  | 1 | 0.796** | 0.887** | 0.970** |
| LDR |  |  | 1 | 0.819** | 0.778** |
| DS |  |  |  | 1 | 0.936** |
| DI |  |  |  |  | 1 |

** Significant at *P*＜0.01.

NDS: Number of diseased spikelets; PDS: Percentage of diseased spikelets; LDR: Length of diseased richides; DS: Disease severity; DI: Disease index.
