# Supplementary material for: Association Mapping and Haplotype Analysis of a 3.1-Mb Genomic Region Involved in Fusarium Head Blight Resistance on Wheat Chromosome 3BS
Source: PLoS One. 2012 Oct 5;7(10):e46444. doi: 10.1371/journal.pone.0046444 (PMC3465345; doi:10.1371/journal.pone.0046444)
Supplement: Table S3 — FHB-related traits of wheat accessions clustered into the same subgroup as Sumai 3 by UPGMA based on a Manhattan dissimilarity matrix. (DOC) [file pone.0046444.s006.doc]

**Table S3 FHB-related traits of wheat accessions clustered into the same subgroup as Sumai 3 by UPGMA based on a Manhattan dissimilarity matrix.**

| Variety name | Origin | NDS | PDS | LDR | DS | DI | PH | FD^a^ | |
| --- | --- | --- | --- | --- | --- | --- | --- | --- | --- |
|  |  |  |  |  |  |  |  | 2009 | 2010 |
| Sumai 3^b^ | Jiangsu | 1.07 | 5.55 | 0.40 | 0.04 | 0.23 | 98.4 | 19 Apr | 2 May |
| Wangshuibai^b^ | Jiangsu | 1.01 | 5.07 | 0.18 | 0.01 | 0.07 | 117.6 | 22 Apr | 5 May |
| Mianyang 11^c^ | Sichuan | 3.35 | 17.51 | 3.25 | 0.35 | 6.22 | 63.13 | 20 Apr | 2 May |
| Ningmai 11^c^ | Jiangsu | 4.41 | 21.05 | 3.78 | 0.36 | 7.50 | 68.10 | 21 Apr | 2 May |
| Aiheshang^*^ | Fujian | 1.10 | 6.61 | 1.33 | 0.13 | 0.85 | 76.7 | 24 Apr | 7 May |
| Duohuangmei^*^ | Jiangsu | 1.39 | 6.73 | 1.54 | 0.18 | 1.36 | 98.7 | 23 Apr | 2 May |
| Liaochun 4^*^ | Liaoning | 1.34 | 6.09 | 2.08 | 0.18 | 1.12 | 109.3 | 25 Apr | 10 May |
| Youzimai | Sichuan | 1.72 | 7.91 | 0.97 | 0.10 | 0.94 | 75.0 | 23 Apr | 2 May |
| Kuikomugi^*^ | Japan | 1.20 | 7.45 | 0.62 | 0.07 | 0.56 | 76.8 | 19 Apr | 27 Apr |
| Sakigakomugi^*^ | Japan | 1.03 | 6.38 | 0.38 | 0.05 | 0.30 | 78.3 | 20 Apr | 27 Apr |

^a^Flowering date of all wheat accessions ranged from 14 April to 27 May, 2009, and 26 April to 30 May, 2010.

^b^Resistant control; ^c^Susceptible control.

^*^Indicates this material contains the same haplotype with Sumai 3.

NDS: Number of diseased spikelets; PDS: Percentage of diseased spikelets; LDR: Length of diseased richides; DS: Disease severity; DI: Disease index; PH: plant height (cM); FD: flowering date.
