# Supplementary material for: Association Mapping and Haplotype Analysis of a 3.1-Mb Genomic Region Involved in Fusarium Head Blight Resistance on Wheat Chromosome 3BS
Source: PLoS One. 2012 Oct 5;7(10):e46444. doi: 10.1371/journal.pone.0046444 (PMC3465345; doi:10.1371/journal.pone.0046444)
Supplement: Table S4 — Comparison of FHB-related traits between the two alleles at umn10 in both European and Asian wheat gene pools. (DOC) [file pone.0046444.s007.doc]

**Table S4 Comparison of FHB-related traits between the two alleles at *umn10* in both European and Asian wheat gene pools.**

| Trait | Allele (bp) | Europe* | | | Asia** | | |
| --- | --- | --- | --- | --- | --- | --- | --- |
|  |  | Min | Max | Mean±S.E | Min | Max | Mean±S.E |
| NDS | 236 | 2.46 | 6.46 | 4.46±0.11a | 1.34 | 6.89 | 4.52±0.13a |
|  | 239 | 3.71 | 5.78 | 4.53±0.15a | 1.03 | 7.58 | 4.52±0.21a |
| PDS | 236 | 11.87 | 34.45 | 21.25±0.56a | 6.09 | 35.38 | 22.65±0.70a |
|  | 239 | 18.42 | 24.54 | 21.58±0.64a | 6.38 | 39.56 | 22.34±1.04a |
| LDR | 236 | 1.94 | 5.78 | 3.72±0.08a | 1.08 | 5.31 | 3.61±0.09a |
|  | 239 | 2.75 | 4.72 | 3.74±0.22a | 0.38 | 5.53 | 3.40±0.14a |
| DS | 236 | 0.19 | 0.51 | 0.34±0.01a | 0.10 | 0.50 | 0.35±0.01a |
|  | 239 | 0.22 | 0.41 | 0.34±0.02a | 0.05 | 0.57 | 0.34±0.01a |
| DI | 236 | 2.34 | 15.52 | 7.72±0.35a | 1.12 | 16.83 | 8.64±0.41a |
|  | 239 | 4.15 | 10.07 | 7.50±0.51a | 0.30 | 20.28 | 8.76±0.61a |

*means 80 contain 236 bp allele and 11 contain 239 bp in European wheat accessions; **shows 100 contain 236 bp allele and 72 contain 239 bp in Asian wheat accessions;

Small letters show significance at *P*＜0.05 when comparing alleles at the same locus for each trait.

NDS: Number of diseased spikelets; PDS: Percentage of diseased spikelets; LDR: Length of diseased richides; DS: Disease severity; DI: Disease index.
